# Editorial: Women in pharmacology of anti-cancer drugs: 2021

**DOI:** 10.3389/fphar.2022.1092204

**Published:** 2022-11-30

**Authors:** Elisa Giovannetti

**Affiliations:** ^1^ Amsterdam UMC, Lab. Medical Oncology, Department Medical Oncology, Cancer Center Amsterdam, Vrije Universiteit Amsterdam, Amsterdam, Netherlands; ^2^ Cancer Pharmacology Lab, Fondazione Pisana per La Scienza, Pisa, Italy; ^3^ EORTC-PAMM Group (Pharmacology and Molecular Mechanisms—European Organization for Research and Treatment of Cancer), Brussels, Belgium

**Keywords:** pharmacology, anticancer drugs, women researchers, oncology, research and diseases, young investigator

For years women have been underrepresented in Science, Technology, Engineering and Mathematics (STEM) university courses and occupations. At present, according to UNESCO, less than 30% of researchers worldwide are women.

Similar statistics have been reported for women working in pharmacological research. A survey of the British Pharmacological Society (BPS) showed that while student members were fairly evenly split between the male and female genders (48% men, 52% women), there was a clear reduction in the numbers of female membership from graduation onwards (25% among Full Members, 15% among Fellows and 8.5% among Honorary Fellows). (https://www.bps.ac.uk/about/our-public-benefit/championing-women-in-pharmacology).

The dropping of women from the pharmacology profession peaked at the mid-career stage, when personal life/family and professional ambitions might conflict. This is in agreement with previous studies describing the “leak” in the academic pipeline for women who decide to leave science across many STEM fields (Ysseldyk et al., 2019). The BPS contacted the female pharmacologists who had left the workplace and asked them why they had taken such decision. The most common reply was the perceived inability to remain competitive in a profession where success is measured by articles published and grants awarded, with little or no consideration given to time away from work because of maternity leave or childcare.

These factors have been amplified during the recent COVID-19 pandemic, when submissions of scientific articles to the medical journals by men has significantly increased, while decreasing for women researchers. Additionally, the number of grant submissions during the first year of the COVID-19 pandemic increased for male but not for female researchers, and female researchers were requesting lower amounts of funds in their applications (Davis et al, 2022).

Against this background, several stakeholders such as governments, professional organizations and foundations are supporting initiative designed to mitigate the impact of COVID-19 on women researchers, with a special focus on junior women academics.

Among the organizations of pharmacological researchers, the Pharmacology and Molecular Mechanisms (PAMM) group of the European Organization of Cancer Research (EORTC) is promoting the young researchers (of note, more than 60% of the PAMM members are women) within the EORTC initiatives for Early Career Investigators, giving opportunities to networking, as well as getting mentorship and funding opportunities.

*Frontiers in Pharmacology* is glad to present a Research Topic to promote the work of women scientists, across all areas of Pharmacology of Anti-Cancer Drugs.

Thanks to the work of contributors, reviewers, and editors, a total of six articles were eventually accepted for publication, including one review and five research articles. These articles reported novel information regarding different topics of anticancer drugs at basic, translational, and clinical levels.

The overview on curcumin and related compounds reported the opposite effects described in multiple tumor types, but also new potential targets in the ubiquitin-proteasome system which broaden the knowledge of the tumor-related effects of these compounds Costantino et al.

New pharmacological approaches against ovarian cancer were evaluated in preclinical studies of the natural drug cinnamaldehyde, which inhibits cancer progression and metastasis by interfering with EGF-induced epithelial-to-mesenchymal transition processes Wang et al. as well as in a meta-analysis on the efficacy and safety of Poria cocos-based formulas in combination with paclitaxel-carboplatin in treating ovarian cancer Peng et al.

A quantitative analysis showed similar efficacy of FDA-approved poly (ADP-ribose) polymerase inhibitors (PARPi) by using progression-free survival data of 1,169 ovarian cancer patients from published randomized trials Gao et al. while a multicenter retrospective study reported patient-associated risk factors for severe anemia in patients with advanced ovarian or breast cancer receiving olaparib monotherapy Tashiro et al.

Lastly, a preclinical study evaluated the potential of cinobufacini injection in preclinical models of triple-negative breast evaluating apoptosis and cycle arrest through the Pin1–TAZ pathway Kong et al.

In conclusion, this Research Topic has provided updated reviews, meta-analysis and new experimental data related to both preclinical, translational and clinical research. Notably, all these articles have been written thanks to the essential contributions of women researchers. However, there is still a lot of scope for recognizing the work of pharmacology’s many female researchers. Such recognition will hopefully improve the work environment and attitudes of the society in their favor. This will bring important life experiences and viewpoints to research questions, methodology and interpretation of results. As the researcher Elana Montagner stated: “if we want to get science out there and improving people’s lives, that needs to have contributions from both men and women”.
